# Excission of Lead Bar

**Published:** 1855-05

**Authors:** John Bell

**Affiliations:** Wapello, Iowa


### EXCISION OF A BAR OF LEAD FROM THE STOMACH—RECOVERY.

BY JOHN BELL, M. D., WAPELLO, IOWA.

On Christmas day, I was summoned to see Mr. L. W. Bates, who, it was said, while performing a favorite feat of running a bar of lead down his throat, had accidentally let it slip, so that it had descended into his stomach ; but before I left my office, he came in followed by a crowd. I asked him if he had swallowed a bar of lead. He said he had ; and that it was nothing wonderful for him to do, as he had swallowed three or four bars at previous times ; this was said in a half waggish manner, and being to all appearance partially intoxicated, and having withal, the reputation of being an expert at juggling and sleight of hand, I supposed it to be one of his tricks, and this opinion was strengthened from the fact that he seemed to be suffering no inconvenience. I came therefore to the conclusion, from the evidence before me, that it was a hoax, but to satisfy myself further, I passed a sound down the œsophagus, but could not discover anything. A few minutes afterwards, Dr. Cleaver and myself, after a brief consultation, concluded to introduce the sound again; we did so, but with no better evidences of the presence of lead than before. We told him to go about his usual employments, and should it trouble him, to send for us. The next day, he went to work and continued at work for three or four days, when, becoming unwell, he went home some six miles from this place, and sent for Dr. Robertson, of Columbus City. On Monday, Jan. 8th, Dr. Robertson requested the physicians of this place to meet him forthwith at the residence of the patient. Dr. Taylor and myself, Dr. Cleaves being absent, answered the summons promptly. Drs. Robertson, Neal, Cleaves, Graham, and Crawford had arrived before us. The patient was closely examined and there was found no perceptible external evidence of the presence of any foreign body in the stomach; the patient was comfortable, up and about, and seemed as well as any of us, if we except some paleness which might have been produced by the regimen enjoined. After considerable conference, it was deemed best not to operate at that time; instructions were given, to keep the patient on a low diet, and open the bowels by a saline laxative, and should any untoward circumstances or symptoms supervene, to notify the physicians at once.

Tuesday, P. M.—Summoned to see Bates immediately. Went, in company with Dr. Taylor; Dr. Robertson soon arrived. Found the patient suffering with considerable gastralgia and abdominal soreness, there had been considerable retching and vomiting of a dark watery fluid; pulse small and tense; great anxiety, restlessness, prostration, and apparent sinking of the vital powers. The bowels had not moved, very sensative to pressure over the left iliac and inguinal region. A consultation was held by the physicians present. It was agreed that a bar of lead had been swallowed and it was also the common opinion that an operation was advisable for its removal; but as night was approaching, it was thought best to defer the operation until the following morning. Sulph. Morph. ¼ gr. as occasion required, to keep him quiet through the night, and fomentations to the bowels.

Wednesday, Jan. 3d—Present, Drs. Robertson, Cleaves, Graham, Taylor and myself. The patient seemed much as on the night previous, great prostration and faintness on attempting to rise.

The patient having been properly placed and secured, chloroform was administered. It produced at first, some nausea and the patient threw up a quantity of black fœtid watery fluid. As soon as insensibility ensued, I made an incision from the point of the second false rib on the left side, to the umbilicus, dividing the skin and cellular membrane, thence through the abdominal muscles, to the peritoneum ; made a minute opening at the lower end of the section, through the peritoneum, passed in the director, and with a probe-pointed bistoury, divided it, through the entire length of the incision. The division of the peritoneum produced a spasmodic contraction of the abdominal muscles, and a large quantity of the omentum and bowels were ejected from the orifice ; these I replaced as speedily as possible, and at once passed my hand inward and upward through the incision, grasped the stomach and immediately discovered the lead and its position. It lay in a direction from right to left, the upper end resting against the walls of the stomach to the right of the cardiac orifice ; the lower end in the greater curvature of the stomach, to the left of and below the pylorus. As it was impracticable to reach the upper end, I seized the bar between my thumb and middle finger, and with the forefinger on the lower end of it, I retracted it upward and backward, for the purpose of making the incision in the stomach as high up as possible. I then passed a scalpel in, along the side of the forefinger as a guide, and divided the coats of the stomach immediately at the end of the bar, making the incision parallel with the muscular fibres, and not larger than to admit of the removal of the lead. I then introduced a pair of long forceps, seized and drew out the lead. The external orifice was closed with the ordinary interrupted suture, and adhesive strips ; a compress was applied, and a roller around the body.

The time of operating was twenty minutes ; considerable delay was occasioned by the protrusion of the contents of the abdomen, which had to be replaced before the operation could proceed. As soon as the effects of the chloroform passed off, $\frac{1}{4}$ gr. Sulph. Morph. was administered, and the patient left in charge of a judicious medical attendant. The following are the the notes of the subsequent treatment of the case :

Wednesday, Jan 9th—During the evening after the operation, the patient was somewhat restless; gave minute portions of Sulph. Morph., which procured intervals of sleep; pulse 85, soft and compressible. Nine o'clock, p. m. became restless; nausea and sinking of the pulse. Melanotic regurgitation. Sulph. Morph. ½ gr.; pulse raised; became full and tense. At this time the salts taken on Tuesday, commenced operating; had seven operations; pulse softened and he dropped into a quiet and refreshing slumber. The patient was kept lying on his back. Twelve, p. m., had a violent attack of vomiting, and threw up about three pints, of a dark greenish fluid, mixed with grumous blood; complains of pain in the stomach and bowels; gave Sulph. Morph. ½ gr., became quiet and slept at intervals until daylight; iced elm water as drink.

Thursday, 10 A. M.—Patient quiet; pulse 85, and moderately full; some thirst, and fever; complains of pain in the stomach and bowels; says he feels a sensation as though water was dropping in his stomach. Morph. continued at regular intervals. Iced toast-water and iced mucilage for drink. Three p. m., pulse 85, rather hard; bled him 10 ℥; continued Morph. and toast-water. Four p. m., quiet, pulse soft. Half-past six, p. m., complains of slight nausea, and has frequent alvine discharges; pulse 86, rather hard; considerable thirst; gave pill, Opii. Ordered Ipecac and Morph.; left powders of Opium and Acet. Plumbi, if the bowels continue moving; toast-water continued.

Friday—Nurse reports a good night's rest; says the pulse ranged, through the night, from 70 to 75; no operation from 8 o'clock till 4 this morning, stools watery, complains of nausea; pulse this morning 83, and soft; tongue white, and rather dry; considerable thirst; slight cough. Five, p. m., found him complaining of gastralgia, nausea and thirst; frequent alvine dejections; pulse 73, hard and full. Nine, p. m., vomited. Gave Morph. and Ipecac; patient became quiet; continue mucilage, discontinue toast-water; repeat powders every three hours.

Saturday, 4 P. M.—Found the patient quiet and easy, pulse 80, soft and natural; tongue clean; an itching sensation in the wound; slight tumefaction, and some soreness of the abdomen; no movement of the bowels since Friday at 4 a. m. Ordered enema.

Sunday, 11 A. M.—Patient comfortable, had two operations from the enema ; raised the bandage and made a small opening through the adhesive strips, for the escape of pus; pulse 77 ; has great desire for nourishment ; may have table-spoonful of peach water every two hours ; directed the bowels to be kept open by enemata. Five, p. m.—Is troubled with melanotic regurgitation ; complains of burning sensation in the superior epigastric region ; pulse 65 soft. Ordered an enema. Pot. Bi-tart. for drink, ¼ gr. Sulph. Morph. as may be necessary.

Monday, 10 A. M.—Patient quiet, pulse 75 full ; face somewhat flushed ; bowels moved once last night ; examined the wound and found it had cicatrized nearly its entire length ; washed and dressed it ; bled him 10 ℥ ; ordered toast water, two table-spoonfuls every two hours ; enema this evening ; Morph., should he be restless during the night.

Tuesday Evening, 6 P. M.—Patient bolstered up in bed and comfortable ; pulse 76. Bowels not moved since 8 last night. An enema at 8 p. m. ; examined the wound and found it doing well. Pot. Bitart. continued.

Wednesday—Found patient quiet; pulse 70 ; rested well through the night ; has an intense craving for food ; face slightly flushed ; increase diet cautiously ; complains of cramp in the extremities on attempting to move.

Thursday—Patient pretty comfortable ; some thirst ; has taken too much, and has exercised more than was prudent. Pulse 75, rather sharp ; face flushed. Washed and dressed the wound, which is healing rapidly. Bowels open. Ordered Morph. ¼ gr., Ipecac gr. 1 ; abstemious diet.

Friday—Patient easy, says he feels well, except some pain in the lower bowels. Pulse 78 and soft, tongue natural ; some tenderness on pressure on the abdomen ; strict antiphlogistic diet.

Sunday—Found the patient standing in the door ; dressed the wound, which looks well. Tongue slightly coated ; bowels inactive; appetite good. Ordered Mass. Hyd. grs. x followed by enema in the morning.

Wednesday, Jan. 17—Found Mr. Bates resting quietly after a walk of half a mile. Washed and dressed the wound ; clipped and removed the sutures, and dressed with Basilicon cerate. With injunctions for bowels to be kept open and care in diet, patient was dismissed. D

It will be observed in this remarkable case that convalescence was established as rapidly as after most of the minor surgical cases. The patient was discharged on the 15th day after the operation and has continued well up to this time; he is now residing in this village, working daily at his trade, that of a shoemaker. The orifice in the stomach was made on the left anterior side, and I think about one inch below the pylorus; the opening was just large enough to withdraw the lead. From some cause, probably from the efforts to vomit, a portion of the omentum had been forced out between the sutures, and when the adhesive strips were removed for the first time, it was found protruding from ½ to ¾ of an inch. Upon examination with a probe, I found it had formed adhesions on both sides of the orifice; I therefore removed the external portion with a pair of scissors.

It may be a matter of surprise, that an operation was not had sooner. Our reply to a question of that nature, is that an operation of that magnitude was not justifiable, as long as there was any doubt as to the lead being in the stomach; and the evening previous to the operation was the earliest time that all doubts of the fact had vanished, and the operation was performed at the earliest practicable moment thereafter. Although I had seen the patient occasionally for three or four days after this singular feat had been performed, and was called on the 8th to witness an operation, during all this time I had not seen one single symptom that was conclusive evidence of the presence of a bar of lead in the stomach.

The length of the bar is 10¾ inches, and its weight 9½ ounces avoirdupois.

WAPELLO, March 15, 1855.

